# PH-binding motifs as a platform for drug design: Lessons from protease-activated receptors; PARs

**DOI:** 10.18632/oncoscience.599

**Published:** 2024-04-25

**Authors:** Jeetendra Kumar Nag, Amnon Hoffman, Chaim Gilon, Beatrice Uziely, Rachel Bar-Shavit

**Keywords:** G-protein coupled receptors (GPCRs), protease-activated receptors (PARs), pleckstrin homology (PH), protease, drug design

While targeted cancer therapy is greatly dependent on specific oncogenic pathways or conferred by genetic alterations, it remains yet challenging and somewhat disappointing. The high level of failure relies on the interplay between the dose for desired therapy versus toxicity, namely the therapeutic index [1]. Furthermore, one should take into consideration resistance response that develops with time to a given drug, contributing immensely to the given drawback. These serious limitations highlight the need for alternative targeting routes.

Although the contribution of G-protein-coupled receptors (GPCRs) in cancer malignancy is growing, GPCR-based therapies are rare. Importantly, GPCRs are involved in many aspects of tumorigenesis, including proliferation, invasion, survival at secondary sites, cancer stemness and cancer associated signaling pathways. Indeed, big data population analyses indicate the significance of GPCRs as valuable targets for therapy in cancer [2]. Protease activated receptors (PARs), a subgroup of GPCRs, form a family of four members; PAR1-4. TCGA and GTEx database show that PAR_2_/*F2RL1* is significantly overexpressed in many types of epithelial malignancies among of which are colon [3], breast [4] and ovarian cancers [5].

The strong connection to tumor biology relies on the abundant presence of proteases residing within the dynamic region of tumor microenvironment either immobilized to the extracellular matrix as a depot storage site, or in a soluble form, all of which are engaged in maintaining tumor growth and progression. This is largely mediated via an active cross-talk with the cell surface receptors aberrantly overexpressed in the neighboring tumor cells. As such is the activation of the PAR oncogene family members (e.g., PAR_1,2&4_), distinctively activated via proteolytic cleavage at their N-terminal extracellular portion and the exposure of cryptic ligands.

We have identified binding motifs within the C-tails of PAR_1,2&4_, indispensable for cancer growth and development [6, 7]. These are the binding sites of pleckstrin homology (PH) domain/s, present in many signal proteins. PAR association with PH- signal proteins initiates a spectrum of intermolecular connections for launching discrete signaling network in cancer [8].

PH domains are preserved protein motifs in diverse signal relaying proteins. They are recognized as versatile modules in protein-protein communication in a plethora of physiological occasions. PH domains are identified by their structural appearances, comprised of C-terminal α-helix and a seven-stranded β-sandwich [8]. Whereas PH domains do not share primary sequence similarities, their folding assembly signifies a predominantly stable structural form engaged in many functions.

We defined binding motifs within the cytoplasmic portion of mammalian PAR_1,2&4_ facilitating selective association with PH domains in Etk/Bmx, AKT and Vav3 as also Gab1 and Sos1. These PH-binding domain sequences are necessary for tumor development as well as physiological placenta anchoring to the uterus decidua. Mutations inserted to either the PAR_1_ PH- binding domain or point mutation at H349A in PAR_2_ or F347L and D349A in PAR_4_, significantly diminished xenograft tumor growth in a mouse model, *in vivo* and of the placental time-restricted anchorage to the uterus decidua in physiological invasion [6, 7].

Backbone cyclization has been demonstrated as an effective tool in the cogent conversion of active regions in proteins to cyclic peptidomimetic drugs [9]. Based on this technology, we selected out of a mini-library, a potent lead backbone cyclic peptide, toward PAR_2_ and PAR_4_ PH-domain termed; P*c*(4-4) of “drug-like” properties. P*c*(4-4) effectively inhibits PAR_2&4_ Akt/PKB associations; PAR instigated Matrigel invasion and migration in vitro and tumor development *in vivo*.

**Scheme 1:**
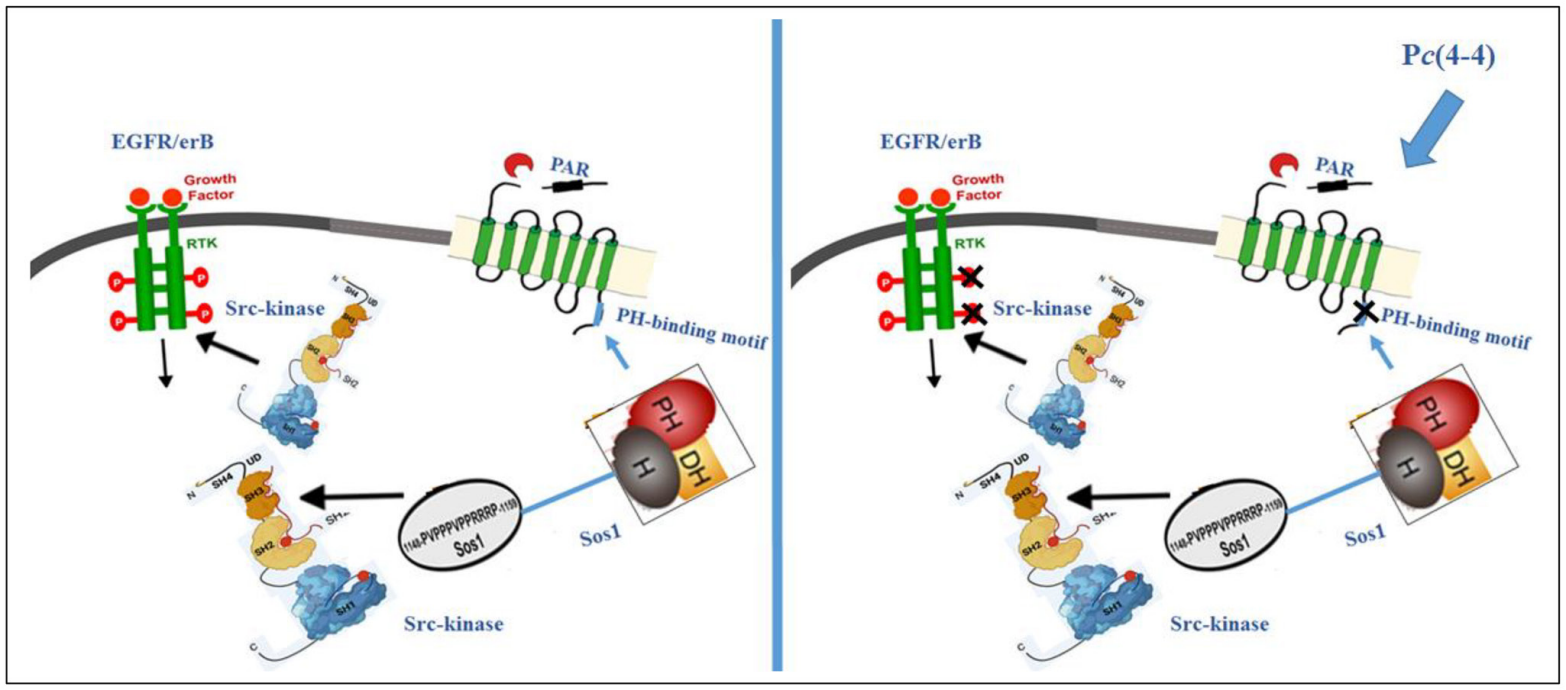
Inside activation of EGFR by PAR as indicated by Tyr-phosphorylation, potently inhibited by P*c*(4-4). PAR activation leads to tyrosine (Tyr)-phosphorylation of EGFR. This is mediated for example, via recruitment the of PH- domain of Sos1 to PAR. Sos1 interact with Src kinase, via its Proline rich region binding to Src SH3 domain. The kinase domain of Src can phosphorylate EGFR C-tail. Application of P*c*(4-4) inhibits association of PH-signal proteins (e.g., Sos1) with PAR. Thereby, P*c*(4-4) abolishes EGFR function (initiated via PARs) as recapitulated via Tyr -phosphorylation.

Such binding motifs offer a prevailing platform system for drug design not only toward PAR directed therapy but also to other cancer driver GPCRs, that potentially possess PH-binding motifs (work in progress). Furthermore, P*c*(4-4) may provide a powerful drug to cancer receptors that can cross-talk and undergo activation by PARs. As such is the EGFR/erbB family (Scheme 1). EGFR/erbB is among the most noticeable cancer targets. It provides a genuine dogma and a follow-up scheme for a future drug design program.

Indeed, AYPGKF peptide ligand activation of PAR_4_ induces EGF receptor (EGFR) Tyr-phosphorylation, which is effectively inhibited by P*c*(4-4). We propose that P*c*(4-4) may serve as a potent drug not only toward PAR-expressing epithelial malignancies but also for treating EGFR/erbB-expressing tumors (7), such as in resistance to traditional therapies. It includes triple negative breast cancer patients as also *Her/Neu* individuals developing resistance to Herceptin.

P*c*(4-4) may serve as a potent drug also toward cancers (e.g., colon, pancreas, stomach, ovary, endometrium, and liver cancers) whereby RNF43 loss of function (LOF) mutations can be detected in a simple blood test. These genetic mutations provide in-fact, a guiding list for the selection of a patient population that will best respond to anti upstream Wnt therapies [10]. Ring finger protein 43 (RNF43) along with zinc and ring finger protein 3 (ZNRF3); RNF43/ZNRF3 are E3 ubiquitin ligases that act to degrade frizzled (FZD) receptors, a subgroup of GPCR. They act to regulate FZDs levels in context and avoid pathological conditions. Wnt ligands activate FZDs and initiate β-catenin stabilization pathway; a core process in cancer. We recently described PAR_2_ as another substrate for RNF43 degradation [11]. LOF mutations in RNF43 facilitates β-catenin stabilization as also the activation of PAR_2_. It is suggested that RNF43 impacts not only on FZDs but acts also on PAR_2_. In cases of RNF43 mutants, these patients can benefit also from PAR directed drugs. Overall, the approach of identifying exclusive target regions within driver GPCRs opens the horizon and provides a straightforward applicable scheme that can be implemented on other principal GPCRs in cancer.
